# Hypoxia‐induced endothelial cell responses – possible roles during periodontal disease

**DOI:** 10.1002/cre2.135

**Published:** 2018-12-14

**Authors:** Reila T. Mendes, Daniel Nguyen, Danielle Stephens, Ferda Pamuk, Daniel Fernandes, Hatice Hasturk, Thomas E. Van Dyke, Alpdogan Kantarci

**Affiliations:** ^1^ Forsyth Institute MA USA; ^2^ Ponta Grossa State University Brazil; ^3^ Faculdade Herrero Brazil; ^4^ Beykent University ‐ Faculty of Dentistry Department of Periodontology Istanbul Turkey; ^5^ Universidade Federal de Santa Catarina Brazil

**Keywords:** endothelial cells, *Fusobacterium nucleatum*, inflammation, oxygen, periodontitis

## Abstract

Background and objective Inflammatory periodontal pockets are known to be hypoxic. Hypoxia influences vascular response to periodontal inflammation, including angiogenesis, which is critical for oxygen and nutrient delivery to periodontal tissues and granulation tissue formation. Our previous work suggests that periodontal bacteria may actively contribute to pocket hypoxia. Herein, we test the hypothesis that *Fusobacterium nucleatum* actively induces low oxygen tension, which modulates angiogenesis and endothelial cell activity. HUVEC cells were incubated in 1.5% oxygen for (Folkman & Shing, 1992)48 hours. Cell proliferation was measured by MTT; surface expression of CD31, CD34 and VEGF receptors (VEGFR1, VEGFR2) were analyzed by FACS. mRNA expression of HIF isoforms, iNOS, eNOS, COX‐2, and VEGF was measured by quantitative PCR. Supernatants were analyzed for the release of IL‐1α, TNF‐α, and VEGF by ELISA or multiplex immunoassays and nitric oxide was measured by colorimetric assay. *F. nucleatum* actively depleted oxygen. Hypoxia resulted in a significant increase of HIF isoforms. iNOS was increased while nitric oxide was unchanged. VEGF release was increased at 4 hours followed by an increase in VEGFR1 at 12 hours, but not VEGFR2. CD31 expression was reduced and CD34 was increased after 48 hours (*p* < 0.05). IL‐1α and TNF‐α release were decreased at 4 hours (p < 0.05), but both increased by 24 hours; TNF‐α increased at 24 h. The data highlight the role of hypoxia in endothelial cell inflammatory changes. *F. nucleatum,* considered a bridging species in the development of periodontopathic biofilms induces hypoxia in the periodontium leading to angiogenic changes in periodontal disease pathogenesis.

## 1 BACKGROUND

Hypoxia and inflammation are closely related (Ahmad & Ahmed, 2004). Hypoxia regulates vascular tone and is a potent stimulus of angiogenesis (Bartruff, Yukna, & Layman, 2005). The increased vasculature enables delivery of oxygen and nutrients to tissues and in healing wounds restores homeostasis (Cochran, 2008). Chronic exposure to hypoxia and changes in angiogenesis are closely related to pathologies including inflammatory diseases, tumors and their metastatic expansion (Cudmore, Hewett, Ahmad, et al., 2012; Darveau, 2010), ischemic diseases (Devine, Marsh, & Meade, 2015) and bone and tissue injuries (Du, Lu, Petritsch, Liu, et al., 2008). Endothelial cell function is central to the interface between the blood and tissues and is the first affected by changes in oxygen tension (Yost, Duran‐Pinedo, Teles, Krishnan, & Frias‐Lopez, 2015). Endothelial cells respond to hypoxia through the expression of regulatory genes mediated by a variety of oxygen dependent signaling cascades (Faller, 1999; Ferrara, Gerber, & LeCouter, 2003). Hypoxia‐inducible factor (HIF) is a key regulator protein of hypoxia‐mediated events (Folkman & Shing, 1992). The HIF family of transcription factors regulates the expression of multiple genes involved in processes that drive adaptation to hypoxia, including cell proliferation, apoptosis, metabolism, immune responses, genomic instability and vascularization (Folkman & Shing, 1992). HIF modulates expression of VEGF, nitric oxide synthases (NOS), and cytokine release regulating angiogenesis (Graves, 2008).

In addition to presenting an environmental change for bacterial colonization, hypoxia may be an important regulator of inflammatory mediators in periodontal disease (Keith, Randal, & Simon, 2011). Very little is known about the impact of hypoxia on oral tissues (Keith et al., 2011). The degree of vascularization depends on the stage of inflammatory disease progression and healing; however, it is not clear how periodontal vasculature responds to hypoxia. The inflammatory process in the periodontium is a dynamic process that is characterized by tissue damage that results from a failure to eliminate the microbial biofilm leading to host‐mediated destructive events (Cochran, 2008; Koo et al., 2007). The pathogenic process in periodontal disease involves an active and reciprocal modification of the host response and biofilm during disease progression. The biofilm expands, evolves, becomes more complex and commensal microbial species become pathogenic as the pathological changes in gingival tissues and periodontal attachment to the tooth surface is destroyed (Lee et al., 2005). As the periodontal pocket deepens, more pathogenic and anaerobic bacteria dominate associated with reduced oxygen in the environment. *Fusobacterium nucleatum* is considered a “bridging species” that is particularly important for the onset and progression of periodontitis because it is thought to enable the colonization of other late‐colonizing periodontopathogenic species such as *Porphyromonas gingivalis (Lin & Sessa,* 2006 *)*.

We have recently characterized the endothelial cell response to *Fusobacterium nucleatum* and demonstrated its active role in promoting proinflammatory changes in endothelial cells, suggesting that the pathogenic progression of periodontitis might be enhanced by modified endothelial cell responses (Liu & Shi, 2012). Based on this observation and literature suggesting that *F. nucleatum* may deplete the oxygen content in its environment (Marsh & Devine, 2011; Mendes et al., 2016), we hypothesized that *F. nucleatum* directly induces hypoxia, which modulates endothelial cell activity in periodontal disease pathogenesis. In order to test this hypothesis, we measured the impact of the *F. nucleatum* on hypoxia and the actions on endothelial cells and their functional regulation.

## 2 MATERIALS AND METHODS

### 2.1 F. nucleatum Growth and Culture

*F. nucleatum* strain ATCC 25586 was cultured on blood agar plates in an anaerobic system under 10% H_2_, 80% N_2_ and 10% CO_2_ for (Tandle et al., 2009)6 days. The cultures were then inoculated into brain‐heart infusion broth, supplemented with hemin, and incubated at 37°C for 2 days until they reached an OD_540nm_ of 0.8, corresponding to 10^9^ CFU mL^−1^. The bacteria were then diluted at 10^7^ CFU mL^−1^ corresponding to a multiplicity of infection (MOI) of 100.

### 2.2 Endothelial Cell Culture

Primary Human Umbilical Vein Endothelial Cells (HUVEC) (ATCC‐PCS‐100‐010) were purchased from American Type Culture Collection (ATCC). Cells were cultured in vascular basal cell medium (ATCC PCS‐100‐030) supplemented with Endothelial Cell Growth Kit‐VEGF (ATCC PCS‐100‐041), penicillin, and streptomycin. Cells were cultured in 75 cm^2^ flasks (Corning®) and maintained in an incubator with 5% CO_2_ at 37°C. Cells were used from passages 4 to 8. Media was changed every three days, in accordance with the manufacturer's recommendations. Cell characterization was accomplished through morphological analysis after reaching confluence. 2 × 10^5^ HUVEC were placed in (Keith et al., 2011)well plates and were pre‐incubated at 37°C for 2 hours. Cells were then incubated in a hypoxia chamber (1.5% O_2_); cells and supernatants were collected and analyzed at baseline up to 48 hours. Normoxia was used as the control for hypoxia conditions.

### 2.3 Oxygen Content in Media in Response to F. nucleatum

In order to measure the oxygen content in cell cultures, 5 × 10^5^ endothelial cells were plated in (Keith et al., 2011)well plates in 1 mL of media. The plates were divided into three groups: control, hypoxia (the plates incubated in a hypoxic chamber) and *F. nucleatum*. Bacteria were added (MOI = 1, 10, 100). The third group was only stimulated with the bacteria (MOI = 1, 10, 100). It was not a co‐culture model and it was incubated in normal conditions of oxygen. Oxygen levels in culture were measured using a NeoFox GT (Ocean Optics Sensors) according to the instructions by the manufacturer. Briefly, 200 μL of each sample was loaded in a 96‐well plate with oxygen sensors, which operate by embedding a ruthenium or platinum‐based porphyrin dye into a sol–gel thin film and correlating fluorescence lifetime to the oxygen partial pressure. The NeoFox Phase Fluorimeter excites this chemistry with a blue LED and collects the resulting fluorescence signal. Measurements were recorded in NeoFox Viewer Software v2.30.

### 2.4 Endothelial Cell Proliferation in Response to Hypoxia

In order to determine the impact of hypoxia on HUVEC cell proliferation, we used the 3‐(4, (Paris et al., 2005)dimethylthiazol‐(Tandle et al., 2009)ul)‐2, (Paris et al., 2005)diphenyltetrazolium bromide (MTT) assay. HUVEC cells were seeded at 1 × 10^4^ cells/well in 96‐well plates and incubated for 24 hours at 37°C. They were then incubated under hypoxia for (Folkman & Shing, 1992)48 hours. At each time point, the media was removed; 90 μL of PBS and 10 μL of MTT solution 5% were added. The plates were incubated for 3.5 hours at 37°C; 75 μL of the solution was removed and 50 μL of dimethyl sulfide (DMSO) was added. The plates were incubated again at 37°C for 10 minutes. Absorbance was measured at 540 nm.

### 2.5 Tube Formation Assay

As an indicator of *in vitro* vascularization, we investigated the role of hypoxia on endothelial cell tube formation. Forty‐five μL of Matrigel (BD Biosciences) were added to each well in 96‐well plates. The plates were incubated at 37°C for 1 hour to allow gelling. Endothelial cells were added at a concentration of 5 × 10^3^ in each well. The plates were then incubated at 37°C for one more hour and incubated under hypoxia. Images were obtained and evaluated on Image‐Pro Plus® Version 4.5.0.29 (Media Cybernetics. Silver Spring, MD, USA). The numbers of tubes were counted and the total area was measured.

### 2.6 Hypoxia‐Inducible Factor (HIF) Expression in Endothelial Cells

In order to study the role of hypoxia on expression of alpha HIF isoforms, which are actively translocated to the cytosol (HIF‐1α, HIF‐2α, and HIF‐3α), endothelial cells were cultured in normoxia and total RNA was extracted using TRIzol reagent. Total RNA was quantified in a spectrophotometer at an absorbance (A) of 260 nm. The RNA samples had an A260: A280 ratio close to 2.0 to guarantee high purity. One μg of total RNA from each sample was subjected to reverse transcription. Each real‐time PCR was carried out in triplicate in a total of 20 μL reaction mixture. Primers used for real‐time PCR analysis were purchased from Life Technologies. The housekeeping gene, glyceraldehyde (Kimmel, Grant, & Ditata, 2016)phosphate dehydrogenase (GADPH), was amplified in each sample as control and was used for normalization. Data analysis was performed using the ΔΔCt method.

### 2.7 Flow Cytometry Analysis of Endothelial Cell Surface Markers

Changes in surface markers due to hypoxia were analyzed by flow cytometry. Endothelial cells were plated in (Keith et al., 2011)well plates (1 mL of media containing 2 × 10^5^ cells) and incubated under hypoxia. Cells were collected, washed twice with PBS/BSA, incubated and labeled with APC anti‐human CD31 antibody (Biolegend), APC anti‐human CD34 antibody (Biolegend), PE anti‐human VEGFR1 antibody (Biolegend) or APC anti‐human VEGFR2 antibody (Biolegend) for 45 minutes, washed twice with PBS/BSA and analyzed by Flow Cytometry (FACScan using CellQuest software, BD Bioscience). Isotype controls for APC and PE (Biolegend) were used. Data were expressed as a percentage of positive cells to target molecules.

### 2.8 VEGF and Pro‐inflammatory Cytokine Release by Endothelial Cells in Response to Hypoxia

The supernatants of endothelial cell cultures were tested for VEGF, IL‐1α, and TNFα using specific MILLIPLEX® Multiplex Assays using Luminex® according to the manufacturer's instructions. Results were corrected to total protein contents of the supernatant media in pg/mL.

### 2.9 NOX release in supernatants, nitric oxide synthase (NOS) and COX2 expression

In order to study the nitric oxide (NOX) release in supernatants, we measured nitric oxide metabolites of nitrite and nitrate using a specific assay kit from R&D Systems (catalog number KGE001) according to the manufacturer's instructions. Results were expressed in μmol/L. Total RNA was quantified as described above for iNOS (inducible NOS), eNOS (endothelial NOS) and COX2 quantification. Each real‐time PCR was carried out in triplicate in a total of 20 μL reaction mixture. Primers used for real‐time PCR analysis were purchased from Life Technologies. The housekeeping gene, glyceraldehyde (Kimmel et al., 2016)phosphate dehydrogenase (GADPH), was amplified in each sample as control and was used for normalization. Data analysis was performed using the ΔΔCt method.

### 2.10 Statistical Analysis

All experiments were performed in triplicate and repeated a minimum of three times. Data are expressed as mean ± SD. Since the distribution of the data did not show major variations among each assay/sample, we have chosen to use the parametric analyses. We analyzed the data using one‐way ANOVA followed by Bonferroni *post hoc* corrections for multiple comparisons. (GraphPad Prism version 5.01, San Diego, CA, USA). *P* < 0.05 was considered significant.

## 3 RESULTS

### 3.1 F. nucleatum Induces Hypoxia

**Figure 1:**
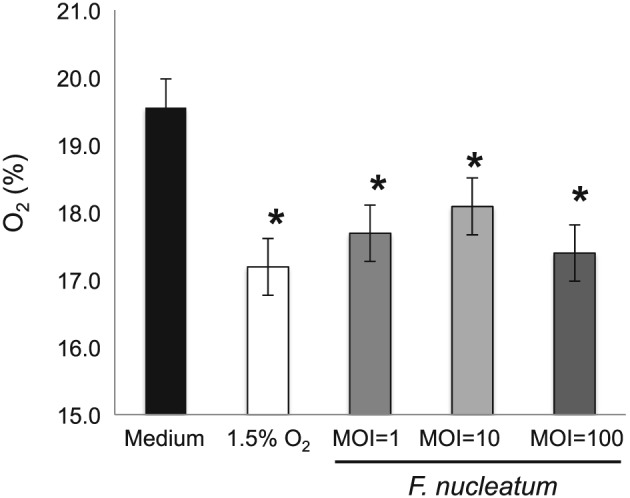
***F. nucleatum* promotes hypoxia.** The oxygen level of samples containing *F. nucleatum* at an MOI 1, 10 and 100 and control (media only) was measured using NeoFox GT (Ocean Optics). *F. nucleatum* significantly reduced oxygen levels in supernatants. Data are expressed as mean ± SD and analyzed using one‐way ANOVA followed by Bonferroni *post hoc*. *P* < 0.05 was considered significant (*p < 0.05)

Endothelial cells were incubated with *F. nucleatum* and compared to a hypoxic atmosphere of 1.5% O_2_ (Figure 1). The data demonstrate that *F. nucleatum* reduces oxygen content creating hypoxia, which is independent of the MOI (*p* < 0.05).

### 3.2 Hypoxia Modulates Proliferation and Tube Formation of Endothelial Cells

**Figure 2:**
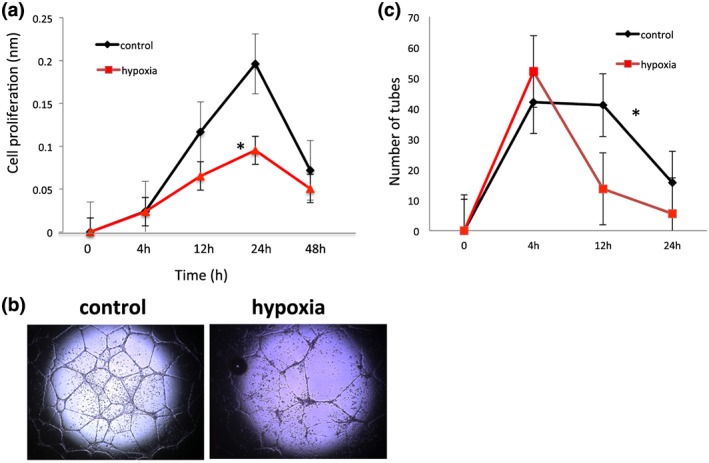
**Hypoxia modulates cell proliferation and tube formation.** Hypoxia significantly reduces endothelial cell proliferation over time (Panel A). Representative images of tube formation after 12 h are shown in panel B. Tube formation is markedly reduced at 12 and 24 hours (Panel C). Data are expressed as mean ± SD and analyzed using one‐way ANOVA followed by Bonferroni *post hoc*. P < 0.05 was considered significant *p < 0.05 compared to control)

The early endothelial proliferation in response to hypoxia was similar to the normoxic control; however, extended hypoxia (24 hours) resulted in significantly suppressed endothelial cell proliferation **(**Figure 2A**)**. In parallel, new vessel formation quantified as the number of tubes was reduced in response to hypoxia and the morphology of the tubes was altered (Figure 2B and C**).**

### 3.3 Hypoxia Modulates Hypoxia Inducible Factor in Endothelial Cells

**Figure 3:**
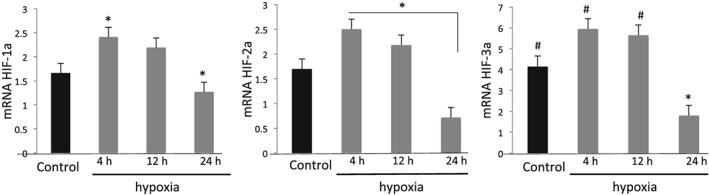
**Hypoxia modulates the Hypoxia Inducible Factor Family of Proteins.** Hypoxia activates HIF isoforms 1α, 2αand 3α as early as 4 hours of incubation. Data are expressed as mean ± SD and analyzed using one‐way ANOVA followed by Bonferroni *post hoc*. P < 0.05 was considered significant (*p < 0.05 compared to control; #p < 0.05 compared to the 24 h group)

Hypoxia results in increased expression of all HIF isoforms after 4 hours of incubation (*p* < 0.05; Figure 3). This impact was transient and resulted in a significant decrease in HIF expression at or below the baseline levels after 24 hours.

### 3.4 The impact of Hypoxia on Adhesion Molecule Expression

**Figure 4:**
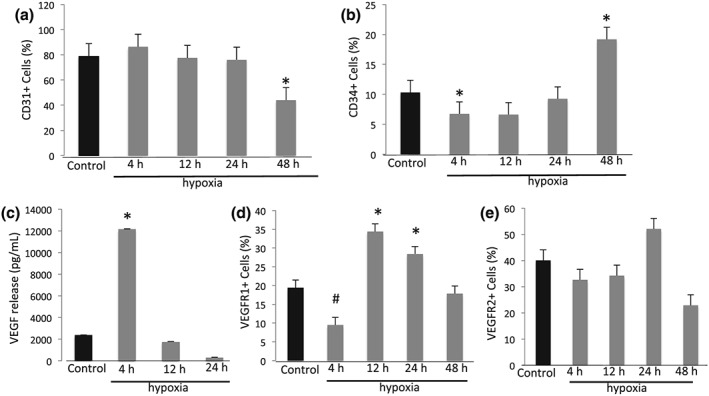
**Hypoxia modulates endothelial cell surface marker expression, changes VEGF release in supernatants and VEGF receptor expression.** The percentage of endothelial cells expressing CD31 (Panel A) or CD34 (Panel B) at baseline and after 4, 12, 24 or 48 hours of hypoxic incubation was analyzed by flow cytometer. Hypoxia increases VEGF release in supernatants (pg/mL) after 4 hours (Panel C) and modulates VEGF receptors 1 (Panel D) and 2 (Panel E) shown as a percentage of expression of endothelial cells at baseline and after 4, 12, 24 or 48 hours of hypoxia. Data are expressed as mean ± SD and analyzed using one‐way ANOVA followed by Bonferroni *post hoc*. P < 0.05 was considered significant (*p < 0.05 compared to control; #p < 0.05 compared to control)

While CD31 expression was constant for 24 hours, there was a reduction after 48 hours (Figure 4A; *p* < 0.05) suggesting reduced cell–cell interaction and an increased vascular permeability. Figure 4B shows a significant decrease in CD34 expression after 4 hours of hypoxia, which increases after 48 hours of hypoxia suggesting increased undifferentiated endothelial cells.

### 3.5 Hypoxia modulates VEGF release and VEGF receptor expression

We next investigated downstream genes targeted by HIF. VEGF release was significantly increased after 4 hours (Figure 4C**;** *p* < 0.05). Further, we quantified VEGF receptor expression by qPCR. VEGFR1 expression was decreased at 4 hours and increased at 12 and 24 hours (*p* < 0.05), returning to initial levels after 48 hours **(**Figure 4D**)**. VEGFR2 expression was stable, with not statistically different changes over the 48‐hour assay (Figure 4E).

### 3.6 Hypoxia Modulates COX‐2 and Up‐regulates iNOS

**Figure 5:**
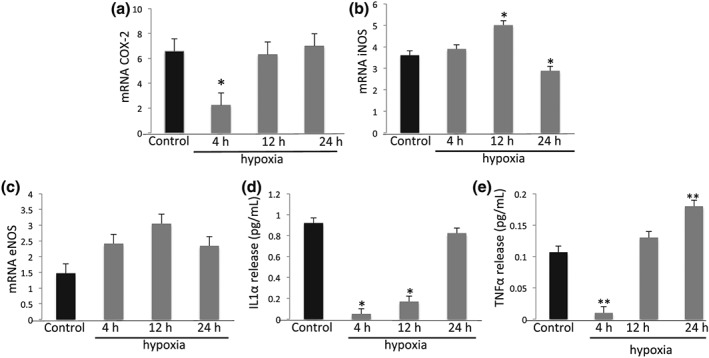
**Impact of Hypoxia on COX‐2 expression, cytokine release, iNOS, and eNOS.** Hypoxia modulates COX‐2 mRNA (shown as fold increase) by endothelial cells (Panel A). After 12 hours iNOS mRNA was higher than after 24 h (Panel B), while eNOS mRNA remained unchanged (Panel C). Hypoxia decreases inflammatory cytokines in supernatants characterized by IL‐1α (pg/mL) (Panel D) and TNF‐α (pg/mL) (Panel E) after 4 hours. IL‐1α returns to basal levels after 24 h, whereas TNF‐α returns to basal levels after 12 h and is increased almost 2‐fold after 24 h. Data are expressed as mean ± SD and analyzed using one‐way ANOVA followed by Bonferroni *post hoc*. P < 0.05 was considered significant (*p < 0.05 compared to control; **p < 0.05 comparing groups 4 h and 24 h)

COX‐2 and NOS play key roles during the endothelial response to inflammation. Expression of COX‐2 and two isoforms of NOS was measured by qPCR. There was an initial and significant decrease in endothelial cell COX‐2 expression after 4 hours of hypoxia, after which the levels returned to normal (Figure 5A; p < 0.05). Nitric oxide release remained unchanged at all time points (data not shown). iNOS was increased after 12 hours (Figure 5B**;** p < 0.05) whereas mRNA for eNOS remained unchanged (Figure 5C), suggesting that only the inducible form of endothelial cell nitric oxide synthase is activated in response to hypoxia.

### 3.7 Hypoxia Modulates Inflammatory Cytokine Release

Hypoxia down‐regulates release of IL‐1α (Figure 5D) and TNF‐α (Figure 5E) in supernatants after 4 hours of incubation. IL‐1α returns to basal levels after 24 h, whereas TNF‐α returns to basal levels after 12 h and is increased almost (Tandle et al., 2009)fold after 24 h. These data suggest that prolonged hypoxia plays a role in progressive inflammation and differentiation of endothelial cells that acquire a pro‐inflammatory phenotype.

## 4 DISCUSSION

Vascular changes are an important aspect of the onset of chronic periodontitis (Olsson, Dimberg, Kreuger, & Claesson‐Welsh, 2006; Zoellnor & Hunter, 1991) as well as other inflammatory lesions, cancer metastases, wound healing, atherosclerosis, tumor growth and angiogenesis (Paris et al., 2005). Hypoxia, which is a master regulator of angiogenesis, indirectly signals VEGF, (Rodriguez‐Morata et al., 2008) and a selective mitogen for endothelial cells (Salim, Nacamuli, Morgan, Giaccia, & Longaker, 2004). Recently, we demonstrated that *F. nucleatum* exerts direct actions on endothelial cells, such as decrease in cell proliferation associated with a reduced tube formation; lower expression of CD31 accompanied by higher expression of CD34 representing an increase in cell permeability and a higher number of undifferentiated cells suggesting that *F. nucleatum* leads to impairment on tissue vascularization during inflammation (Liu & Shi, 2012). Based on this work, we hypothesized that *F. nucleatum* directly contributes to a hypoxic environment modulating endothelial cell function in the pathogenesis of periodontal disease. Collectively, the data suggest that *F. nucleatum* can impact the endothelial cell function directly and indirectly through the hypoxic changes in the environment.

Human endothelial cells incubated under hypoxic conditions demonstrated reduced proliferation and suppressed and irregular angiogenesis mediated by HIF isoforms. Downstream targets of HIF including iNOS activation, VEGF release, and VEGFR1 appear to mediate these changes that were associated with an endothelial cell pro‐inflammatory phenotype shift. The data demonstrate that prolonged hypoxia results in endothelial dysfunction and increased inflammation. In addition, functional changes of endothelial cells were characterized by alterations in adhesion molecules (CD31 and CD34) that are associated with a loss of cell–cell adhesion and a significant increase in the number of undifferentiated cells.

Endothelial cell proliferation was decreased with reduced tube formation suggesting defective and disorganized vascular activity, which may be a critical characteristic of inflammatory vascularization. A previous study showed that a (Sluimer, Gasc, van Wanroji, et al., 2008)hour exposure to hypoxia stimulated cell proliferation (Ferrara et al., 2003). We did not measure the (Sluimer et al., 2008)hour time point but our closest data point at 4 hours showed no difference between normoxic and hypoxic conditions. We observed a stable hypoxia‐mediated response as of (Keith et al., 2011) hour time point, which lasted throughout the observation period. This finding suggests that a “chronic” exposure to hypoxia is critical for changes in cell function. Persistent hypoxia also changed the expression of surface markers on endothelial cells linked to function. Decreased expression of CD31 and an increase in CD34 suggests an increased potential for vascular permeability of undifferentiated cells, both of which are critical signs of inflammation. CD31 serves as an adhesion molecule on endothelial cells (Sluimer et al., 2008) and is involved in the regulation of leukocyte detachment, T‐cell activation (Tandle et al., 2009; Taylor, 2008), and platelet activation and angiogenesis (Taylor, 2008). CD31 activation may be dependent on inflammatory cytokines (Taylor, 2008) and a reduction in CD31 expression may be associated with increased T cell activation (Tandle et al., 2009). These findings are consistent with the chronicity of inflammation induced by hypoxia and implicate endothelial cells as playing an active role in the induction of inflammation.

Hypoxia had a direct impact on VEGF expression and actions promoting a switch in the VEGFR1: VEGFR2 ratio, as well as an initially increased VEGF‐A release. Numerous studies emphasize that VEGF and its receptors are critically important regulators of endothelial cell and vessel formation (Ferrara et al., 2003; Torimoto, Rothstein, Dang, Schlossman, & Morimoto, 1992). Under physiological conditions, endothelial cells express approximately 10 times less VEGFR1 than VEGFR2 (Unger, Krump‐Konvalinkova, Peters, & Kirkpatrick, 2002; Ve Val & Black, 2009). VEGFR1 mainly modulates VEGF activity creating heterodimers with VEGFR2 (Wigerup, Påhlman, & Bexell, 2016). VEGFR1 negatively regulates cell proliferation through VEGFR2, and its increase reduces angiogenesis (Wirthlin & Hussain, 1992). Here, we demonstrate that early hypoxia reduces VEGFR1 expression, but this is reversed and increased over time returning to baseline by 48 hours. These observations are consistent with our observed decrease in cell proliferation induced by hypoxia but it may also highlight a limitation of the in vitro testing of exposure to hypoxia and hypoxia‐mediated events.

The most striking alteration induced by hypoxia was the transcriptional activation of the Hypoxia Inducible Factor (HIF) family of proteins as early as 4 hours after exposure to hypoxia. HIFs coordinate the first steps in vessel development through a paracrine mechanism. HIF activation correlates with the presence of more differentiated cells (Zhao et al., 2012; Zoellnor & Hunter, 1991), which taken together with our findings, suggest that CD34‐mediated differentiation of endothelial cells was regulated by HIF in response to hypoxia. HIF‐mediated signaling is linked to VEGF generation and inflammatory cytokine production (Folkman & Shing, 1992). Endothelial cells showed a (Sluimer et al., 2008) fold increase in VEGF with hypoxia in 4 hours followed by TNF‐α increase suggesting a phenotypic change in endothelial cells towards becoming more pro‐inflammatory.

In summary, the data highlights the role of hypoxia in the induction of endothelial cell inflammation that can be induced by *F. nucleatum,* a periodontal organism long thought to be involved in the development of biofilm dysbiosis. The shift between normoxia‐hypoxia and different gradients of oxygen content at any given time and any site in the periodontal pocket and biofilm is highly dynamic and very complex. Our study addresses only an angle of this dynamic system, which may be mechanistically relevant to solve this complexity. There are obvious limitations of *in vitro* studies and reductionist approaches using cell culture systems and artificial environments. Yet, the demonstration of the direct actions of *F. nucleatum* taken together with elucidation of downstream impact on endothelial cell phenotype and function may provide an important mechanistic insight for our understanding of hypoxia‐induced angiogenic changes in periodontal disease pathogenesis.

## CONFLICT OF INTEREST

The authors declare no conflicts of interest.
